# Multimode fiber tip Fabry-Perot cavity for highly sensitive pressure measurement

**DOI:** 10.1038/s41598-017-00300-x

**Published:** 2017-03-23

**Authors:** W. P. Chen, D. N. Wang, Ben Xu, C. L. Zhao, H. F. Chen

**Affiliations:** College of Optical and Electronic Technology, China Jiliang University, Hangzhou, China

## Abstract

We demonstrate an optical Fabry-Perot interferometer fiber tip sensor based on an etched end of multimode fiber filled with ultraviolet adhesive. The fiber device is miniature (with diameter of less than 60 μm), robust and low cost, in a convenient reflection mode of operation, and has a very high gas pressure sensitivity of −40.94 nm/MPa, a large temperature sensitivity of 213 pm/°C within the range from 55 to 85 °C, and a relatively low temperature cross-sensitivity of 5.2 kPa/°C. This device has a high potential in monitoring environment of high pressure.

## Introduction

Optical fiber Fabry-Perot (FP) interferometric pressure sensors have been attractive for a wide range of applications in biomedicine, healthcare, civil engineering, and automotive and aerospace industries^1^. Especially the FP cavity at the fiber tip can provide a convenient reflection mode of operation.

There are two types of optical fiber FP interferometer (FPI) pressure sensors, operated on cavity length change^2–9^ or dominated by cavity refractive index (RI) variation^10–14^, respectively. Many FPI sensors operated on cavity length change have relatively low pressure sensitivity except those based on thin diaphragm^2–5^. One of the key elements to determine the pressure sensitivity of the FPI is the thickness of the diaphragm employed. A thin diagram has a relatively high sensitivity. However, the mechanical strength of a thin diagram is rather poor as the thin diaphragm attached at the fiber end is fragile and easily to be cracked. Moreover, the FPI sensors based on a thin diagram exhibit a small measurement range, typically in a few tens of kPa^4, 5^. Several FPI configurations operated on cavity RI variation have been developed for gas pressure sensing. For instance, an FPI tip based on side-hole dual-core photonic crystal fiber (PCF) exhibits a measurement range from 0 to 500 MPa and a sensitivity of 32 pm/MPa^12^. Other FPI tip sensors based on twin-core PCF or fiber Bragg grating (FBG) also have similar performance^13, 14^. Although the FPI sensors operated on cavity RI variation usually have a large measurement range and good robustness, their pressure sensitivities are relatively low, typically in a few tens of pm/MPa^11–14^, unless a carefully designed sensor head is utilized^15^, which increases the device complexity and the fabrication difficulty.

Another key element that determines the pressure sensitivity of the FPI operated on cavity length change is the size of the diaphragm employed^16^, and the larger the size, the higher the sensitivity that can be achieved. In general, the size of diagram is limited by the diameter of the fiber core of single mode fiber (SMF), which is less than 9 μm. By contrast, the core size of multimode fiber (MMF) is more than 60 μm. Moreover, more sensitivity enhancement would be expected if the diaphragm could utilize the whole diameter of MMF.

Here we propose and experimentally demonstrate a new type of optical fiber tip FPI pressure sensor based on an etched end of MMF filled with ultraviolet (UV) adhesive. Because of the large size of tapered hole formed in the MMF and the large elasticity coefficient of UV adhesive, the fiber tip FP cavity is compact in size, robust in structure, simple in fabrication, and convenient in operation. The gas pressure sensitivity achieved within the measurement range between 0 and 1 MPa (limited by the pressure meter we used) is −40.94 nm/MPa. The temperature sensitivity of the device is 213 pm/°C, within the range from 55 to 85 °C, which gives a relatively low temperature cross-sensitivity of 5.2 kPa/°C. Moreover, the device can also be used for RI sensing with a sensitivity of ~−73.54 nm/RIU (RI unit) within the range from 1.332 to 1.372, which shows its versatile measurement capability.

## Device Fabrication and Principle

**Figure 1:**
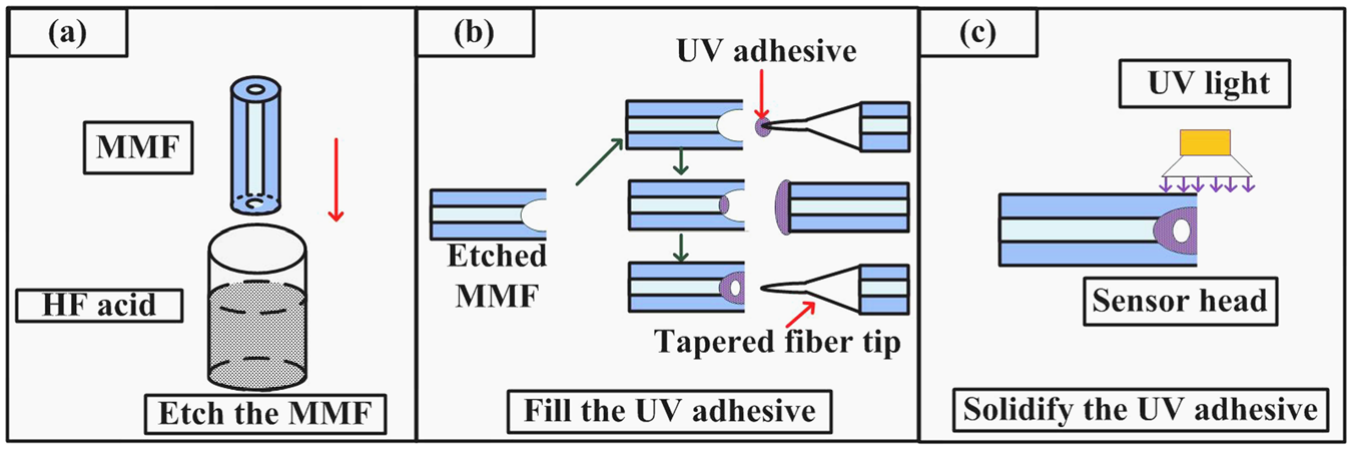
Schematic diagram of the fabrication process of the optical fiber sensor head. (**a**) A taper-shaped hole at the end of MMF is formed by HF etching. (**b**) The taper-shaped hole at the end of MMF is filled with UV adhesive to form an inner air-cavity. (**c**) The UV adhesive is solidified.

The optical fiber FP cavity sensor head is composed of an etched MMF filled with UV adhesive. During the sensor head fabrication process, the end face of MMF with a core diameter of 62.5 μm and a nominal effective RI of 1.4682 (at 1550 nm) is etched by use of hydrofluoric (HF) acid to form a tapered hole cavity. The UV adhesive (Norland, NOA68) employed can sustain the temperature change from −80 °C to 90 °C, and has the RI of 1.54 (at 1550 nm). The fabrication process of the device is illustrated in Fig. 1.

Initially, a section of MMF with length of ~20 cm is immersed into HF acid with the concentration of 40% and the immersed length is ~0.25 cm as shown in Fig. 1(a). The etching process takes ~10 minutes before a taper-shaped hole of several tens micrometers in depth is formed at the end face of the MMF. Next, the taper-shaped hole is filled with UV adhesive by use of following steps as illustrated in Fig. 1(b):

1. UV adhesive attached on the tip of a tapered optical fiber is filled into the taper-shaped hole.
2. UV adhesive attached on the flat end of an MMF is used to seal the taper-shaped hole and form an inner air-cavity due to the remaining air in the taper-shaped hole region.
3. The shape and position of the inner air-cavity can be controlled by using a tapered fiber tip to push the UV adhesive at appropriate position, while observing the reflection spectra of the device via an optical spectrum analyzer (OSA).

Finally, the UV adhesive is solidified by use of a UV light as shown in Fig. 1(c), to provide a robust structure of the device.

The size of inner air-cavity can also be controlled by adjusting the usage of UV adhesive used. For instance, if a small inner air-cavity is needed, less UV adhesive should be used.

**Figure 2:**
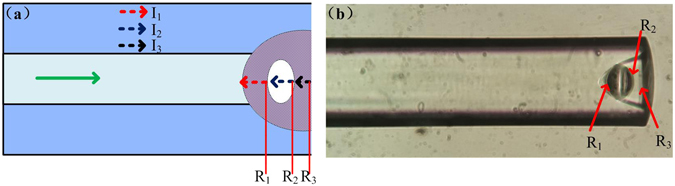
(**a**) Schematic diagram and (**b**) the microscope image of the sensor head.

The schematic diagram and the microscope image of the sensor head are shown in Fig. 2. There are three main reflection surfaces in the device sensor head, at the interfaces of adhesive-air (R_1_), air-adhesive (R_2_) and adhesive-air (R_3_), respectively, which form three FP cavities.

The two reflection surfaces of R_1_ and R_2_ create an FP cavity (C_1_). When light beam traveling along the MMF arrives at R_1_, part of the light is reflected back, and the rest continues to propagate until reaching R_2_ and experiencing partial reflection. The light beam transmitted through R_2_ finally arrives at R_3_ and is partially reflected again. The two reflection surfaces of R_2_ and R_3_ thus forming another FP cavity (C_2_). Meanwhile, the two reflection surfaces R_1_ and R_3_ can also form an FP cavity (C_3_) with a cavity length that is equal to the sum of cavity lengths of C_1_ and C_2_.

**Figure 3:**
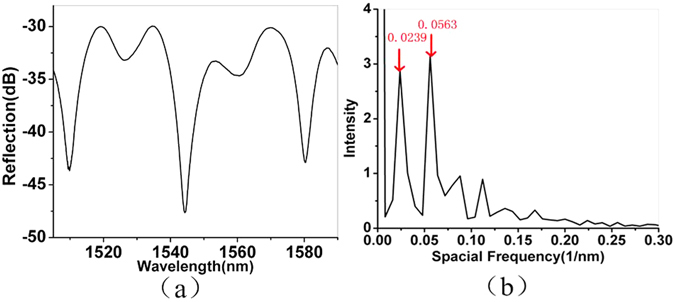
(**a**) Reflection spectra of the device sample. (**b**) Spatial frequency spectra of the device sample.

The reflection spectrum of the device sample fabricated and its corresponding spatial frequency spectrum obtained by use of fast Fourier transform are displayed in Fig. 3. It can be observed from the figure that there are two dominant side peaks in the spatial frequency spectrum, located at ~0.0239 and ~0.0563 nm^−1^, respectively, which indicates an interference pattern of two FP cavities.

The well-known expression of free spectral range (FSR) is written as

1 $$\mathrm{FSR}={\lambda }^{{\rm{2}}}/2{\rm{nL}}$$

where λ is the wavelength, n is the RI of the cavity, and L is the cavity length. As the RI of UV adhesive is 1.54 and the cavity lengths of C_1_ and C_2_ are ~37.7 μm, ~18.7 μm, respectively, the cavity length of C_3_ is the sum of the length of C_1_ and C_2_, i.e., 56.4 μm. By taking the wavelength of 1550 nm, the spatial frequency peak positions for three FP cavities C_1_, C_2_ and C_3_ can be determined as ~0.0314, ~0.0239 and ~0.0554 nm^−1^, respectively. This reveals that C_2_ and C_3_ are the dominant FP cavities as the experimentally obtained results agree well with those obtained from the calculations. Thus, the output spectrum of the device consists of two superimposed FP spectra of C_2_ and C_3_.

The output light intensity (I) of FP cavity C_2_ and C_3_ can be written as

2 $${\rm{I}}={{\rm{I}}}_{{\rm{1}}}+{{\rm{I}}}_{{\rm{2}}}+2{{\rm{I}}}_{{\rm{3}}}+2\sqrt{{{\rm{I}}}_{{\rm{2}}}{{\rm{I}}}_{{\rm{3}}}}\,\cos \,{{\rm{\Phi }}}_{{\rm{23}}}+2\sqrt{{{\rm{I}}}_{{\rm{1}}}{{\rm{I}}}_{{\rm{3}}}}\,\cos \,{{\rm{\Phi }}}_{{\rm{13}}}$$

where I_1_, I_2_, and I_3_ are the light intensity reflected from adhesive-air surface R_1_, air-adhesive surface R_2_, and adhesive-air surface R_3_, respectively, and Φ_23_ and Φ_13_ are the introduced phase shifts of the cavity C_2_ and C_3_, respectively, given by

3 $${{\rm{\Phi }}}_{23}=\frac{4{{\rm{\pi }}{\rm{n}}}_{{\rm{2}}}{{\rm{L}}}_{{\rm{2}}}}{\lambda }$$

4 $${{\rm{\Phi }}}_{{\rm{13}}}=\frac{4{\rm{\pi }}({{\rm{n}}}_{{\rm{1}}}{{\rm{L}}}_{{\rm{1}}}{+{\rm{n}}}_{{\rm{2}}}{{\rm{L}}}_{{\rm{2}}})}{\lambda }$$

where n_1_ and n_2_ are the RI of air, UV adhesive, L_1_ and L_2_ are cavity length of C_1_ and C_2_, respectively.

**Figure 4:**
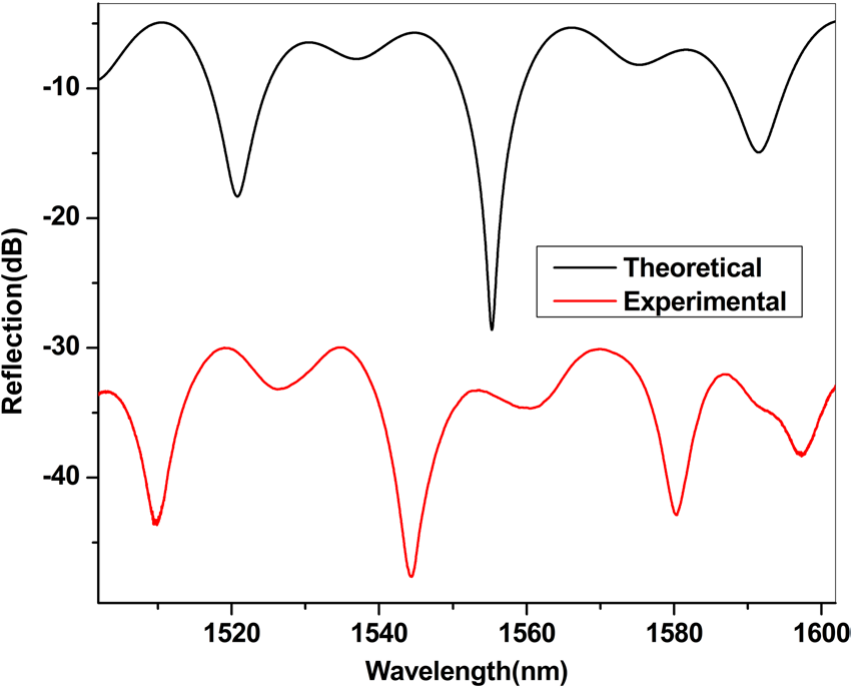
Reflection spectra of the FP cavity device obtained from theoretical simulations and experiment.

From Eqns (2)–(4), by taking n_1_ = 1, n_2_ = 1.54, and the reflection coefficient of the surface of R_1_, R_2_ and R_3_ as (n_1_ − n_2_)^2^/(n_1_ + n_2_)^2^ = 0.0452, the reflection spectrum of the FP cavity device can be simulated and the result obtained is displayed in Fig. 4, together with that obtained from the experiment as shown previously in Fig. 3(a), to facilitate the comparison. It can be seen from Fig. 4 that the two reflection spectra have nearly the same waveform, and their intensity difference comes from the insertion loss of the device while the wavelength shift existed in the experimental fringe pattern is likely due to the initial phase of the fringe pattern and the dispersion effect of the optical fiber.

## Experiments and Discussions

**Figure 5:**
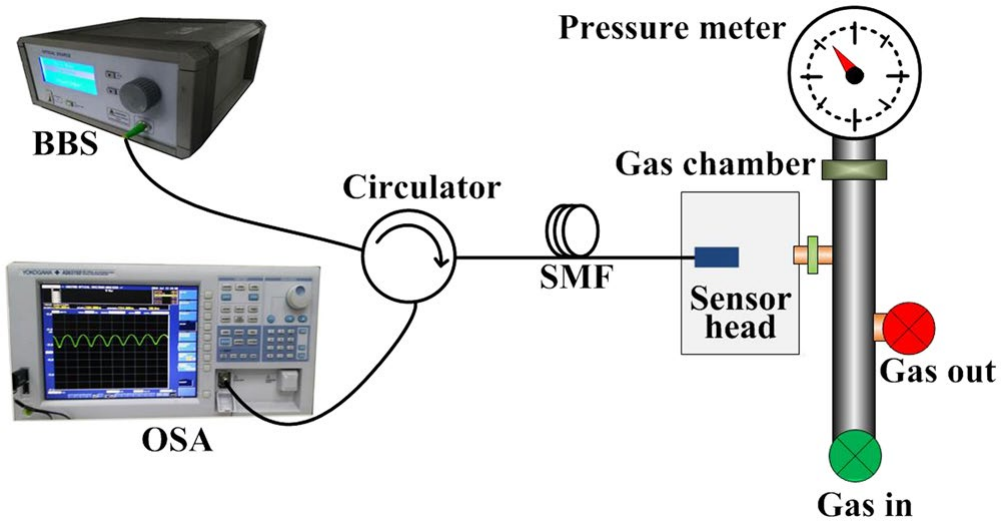
Schematic diagram of experimental setup.

The experimental setup used to test the response of FP sensor head to gas pressure is displayed in Fig. 5. Light output from a broadband light source (BBS) is introduced to the FP sensor head via a circulator. The sensor head is placed in a gas chamber, where the gas pressure can be adjusted by use of an air pump (Wisdom Billiton, Y039), measured by a pressure meter (ZHITUO, YB-150). The sensor output is directed to an optical spectrum analyzer (OSA) with the resolution of 0.05 nm to monitor the spectrum.

**Figure 6:**
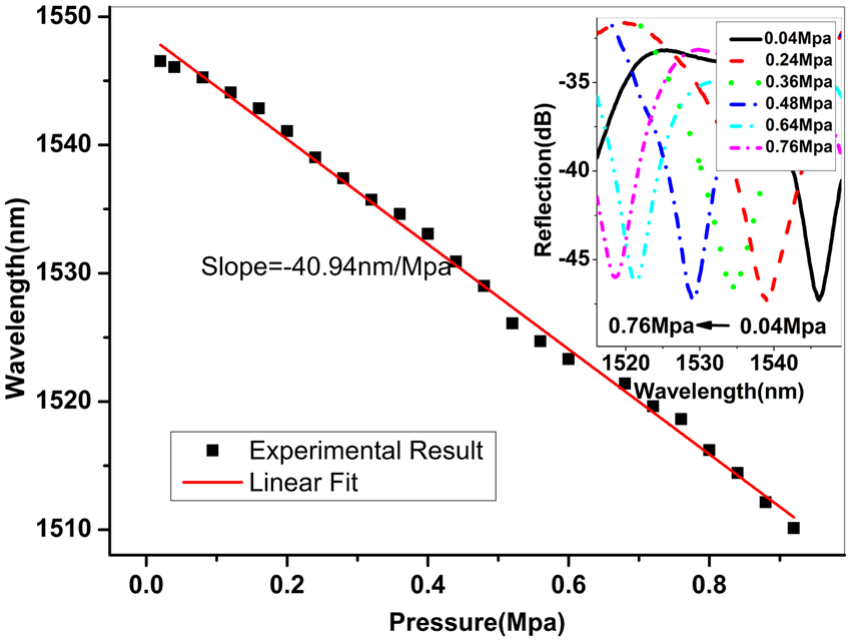
Dip wavelength shift with the increase of pressure, and the inset shows the reflection spectra of the device at different pressures.

In the pressure measurement, the fiber in the chamber was kept in a straight line to avoid any bending-induced effects. The pressure was increased from 0.02 to 0.04 and then to 0.92 MPa with a step of 0.04 MPa, and the reflection spectrum was monitored in real-time by use of an OSA. Figure 6 and its inset demonstrate the shift of dip wavelength in the reflection spectrum with the pressure variation. It can be seen from the figure that the dip wavelength is decreased with the increase of pressure values from 0.02 to 0.92 MPa, and a good linear relationship can be obtained. The pressure sensitivity achieved is −40.94 nm/MPa.

**Figure 7:**
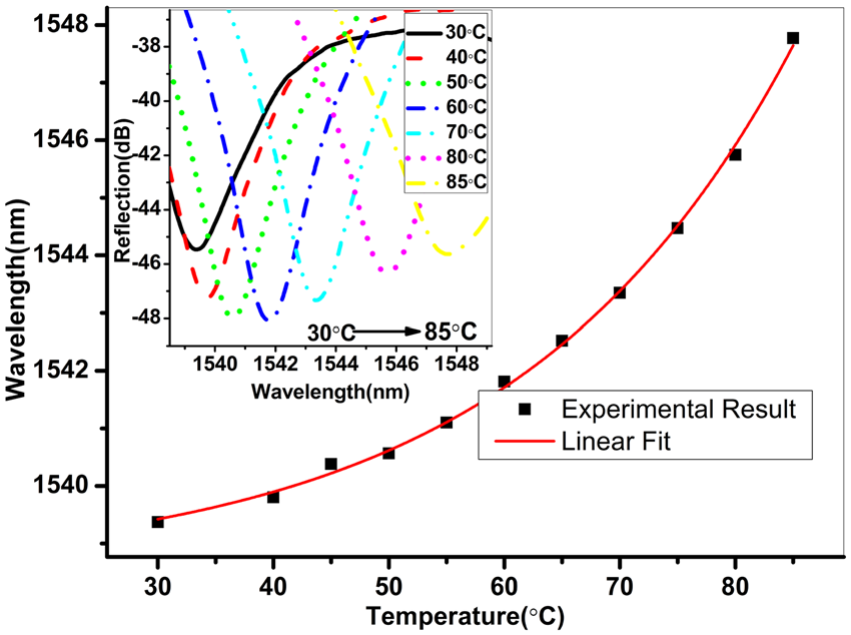
Fringe dip wavelength shift with the temperature variation. Inset shows the reflection spectra of the device at different temperatures.

The temperature effects on the fiber sensor device were investigated by placing the sensor head in an electrical oven and gradually increasing the temperature from room temperature to 85 °C with a step of 5 °C. The temperature was maintained for 10 min at each step to make sure the temperature in the chamber was stabilized. The dip wavelength shift with the temperature variation is demonstrated in Fig. 7, where the inset shows the reflection spectra at different temperatures. A fringe dip near ~1540 nm at the temperature of 30 °C is found to experience a red shift with the increase of temperature. The highest sensitivity obtained is ~213 pm/°C within the temperature range from 55 °C to 85 °C. However, considering of the pressure sensitivity of −40.94 nm/MPa obtained in the experiment, the temperature cross-sensitivity is calculated to be only 5.2 kPa/°C, which is much smaller than that of the sensors based on side-hole dual-core PCF^12^ (1 MPa/°C) and on FBG in the SMF (2.3 MPa/°C)^14^.

**Figure 8:**
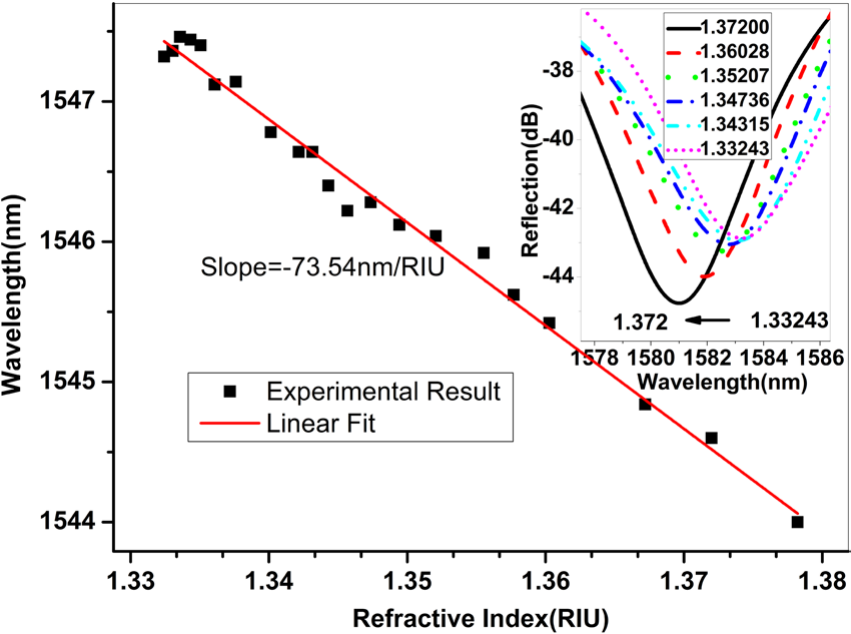
Dip wavelength shift with RI, and the inset shows the reflection spectra of the device at different RI values.

The device can also be used for RI sensing. In the experiment, the device sample was immersed into a series of RI liquids and the reflection spectra recorded had a resolution of 0.05 nm. Each time after the liquid sample was measured, the fiber sensor head was rinsed with water carefully until the original spectrum (i.e., the reference spectrum) could be restored and no residue liquid was left on the sensor head surface. Figure 8 shows the interference fringe dip wavelength shift with the RI change and the sensitivity of ~73.54 nm/RIU was achieved. In the inset of Fig. 8, the wavelength variation as a function of RI is plotted.

Currently, the pressure measurement range achieved in the experiment is limited by the air pump used, which only provides a pressure value up to 1 MPa. However, our device has the potential of achieving much higher pressure measurement range due to its robust structure. As a number of wavelength dips exist in the reflection spectrum as shown in Fig. 3(a), and the device is sensitive to a range of physical parameters, a simultaneous multiple parameter measurement can be expected.

## Conclusion

To summarize, we have proposed and fabricated an optical fiber FP interferometer by use of etched MMF filled with UV adhesive. The gas pressure variation induces the air-cavity length change, which causes the change in optical path difference of the FPI, and in turn leads to the reflection spectrum shift. The sensor device exhibits a high pressure sensitivity of −40.94 nm/MPa and a good temperature sensitivity of 213 pm/°C within the range from 55 °C to 85 °C, and a RI sensitivity of ~−73.54 nm/RIU within the range from 1.332 to 1.372. The temperature cross-sensitivity of the device is 5.2 kPa/°C. Such a device is based on low cost MMF, compact in size, robust in structure, simple in fabrication, convenient in operation, which makes it highly attractive for pressure sensing.
